# Genetic variation among sea turtle life stages and species suggests connectivity among ocean basins

**DOI:** 10.1002/ece3.9426

**Published:** 2022-10-30

**Authors:** Katrina F. Phillips, Katherine R. Martin, Gustavo D. Stahelin, Anna E. Savage, Katherine L. Mansfield

**Affiliations:** ^1^ Department of Biology University of Central Florida Orlando Florida USA

**Keywords:** *Chelonia mydas*, dispersal, gene tree, mitochondrial DNA, mixed stock analysis

## Abstract

Regional genetic differentiation of mitochondrial lineages occurs in migratory species with natal philopatry such as sea turtles. However, early juvenile dispersal represents a key opportunity for gene flow and colonization of new regions through founder events, making it an important yet under‐studied life stage. To assess connectivity among sea turtle life stages and ocean basins, we sequenced mitochondrial DNA (mtDNA) fragments from 35 juveniles sampled in the Gulf of Mexico from the rarely observed dispersal stage across three species: green turtles (*Chelonia mydas*; *n* = 30), hawksbills (*Eretmochelys imbricata*; *n* = 3), and loggerheads (*Caretta caretta*; *n* = 2). We estimated green turtle rookery contributions using a many‐to‐many Bayesian mixed stock analysis that incorporated dispersal probabilities based on rookery size and transport via ocean currents. We assembled a gene tree including 709 distinct mtDNA control region haplotypes from the literature for all seven extant sea turtle species to assess gaps in life‐stage data across ocean basins, as well as contextualize the lineages we sampled from dispersing juveniles. Our results indicate a high likelihood that green turtles sampled in the Gulf of Mexico originated from rookeries along the coast of Mexico, with smaller contributions from Costa Rica and Suriname. The gene tree analysis yielded species‐level relationships consistent with those presented previously, while intra‐species relationships between lineages and ocean basins differed, particularly within loggerhead and green turtle clades. Our results highlight the lack of genetic data from juvenile sea turtles, especially the early dispersal stage, and the potential for these data to answer broader questions of connectivity and diversification across species and lineages.

## Graphical abstract

Regional genetic differentiation based on mitochondrial DNA (mtDNA) occurs in migratory species with natal philopatry such as sea turtles. To assess connectivity among sea turtle life stages and ocean basins, we (1) genotyped juveniles sampled in the Gulf of Mexico from the rarely observed dispersal stage; (2) estimated rookery contributions using a many‐to‐many Bayesian mixed stock analysis; (3) assembled a gene tree based on 709 long‐fragment mtDNA haplotypes from the literature for all seven extant sea turtle species to assess gaps in life stage data across ocean basins and characterize the lineages we sampled from dispersing juveniles. Our results highlight the lack of genetic data from juvenile sea turtles, especially the early dispersal stage, and the potential for these data to answer broader questions of connectivity as well as diversification across species and lineages.

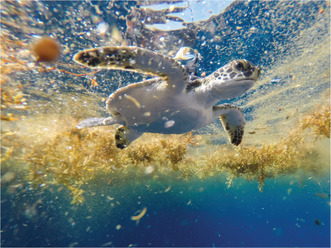

## 1 INTRODUCTION

Juvenile dispersal distributes offspring across habitats, which may reduce predation or competition for limited resources (Forero et al., 2002; Zhuang et al., 2002) while promoting gene flow and recruitment to new habitats (Bohonak, 1999; Howard, 1960). Recruitment away from the natal site may be especially important in patchy environments where home ranges are restricted in size (Barlow, 1981). Broadscale juvenile dispersal also promotes resilience over evolutionary timescales, as a distribution of juveniles across regions increases the potential for species recovery from acute and localized habitat disturbances, as well as long‐term perturbations such as climate change (Bowen et al., 1994; Howard, 1960; Shamblin et al., 2014).

Juvenile dispersal is common in marine environments, where ocean currents facilitate movement among planktonic invertebrates (Baums et al., 2006; Duffy, 1993; McMillan et al., 1992), larval fish (Doherty et al., 1995; Waples, 1987), and young sea turtles (Putman & Naro‐Maciel, 2013). In migratory species like sea turtles and salmonids, juvenile dispersal is more complex in that early dispersal is later followed by natal philopatry that assures mature females reach viable nesting or spawning habitat (Brothers & Lohmann, 2015; Lohmann et al., 2008; Putman et al., 2010). This site fidelity reduces potential gene flow and reinforces spatial patterns in mitochondrial lineages (Bowen et al., 1994; Bowen & Karl, 2007).

The least‐studied sea turtle life stage is the initial post‐hatching dispersal stage, lasting 1–12 years, after which most species recruit to juvenile habitats generally closer to the coast (Bolten, 2003; Mansfield & Putman, 2013). Known as the ‘lost years’, individuals in the early dispersing stage travel tens to thousands of kilometers from their natal rookery (Mansfield et al., 2014, 2021; Putman & Mansfield, 2015; Putman & Naro‐Maciel, 2013; Shamblin, Witherington, et al., 2018). Connectivity among juvenile and mature habitats needs to be assessed to effectively manage conservation priorities across the life cycle, as frequencies of maternally inherited mitochondrial DNA (mtDNA) haplotypes within and among rookeries are used to delineate distinct population segments and regional management units for these turtle species of conservation concern (Wallace et al., 2010). From an evolutionary perspective, juvenile dispersal is a valuable proxy for understanding how species initially colonized ocean basins (Jensen et al., 2019; Reis et al., 2010; Shamblin et al., 2014) and provides insight into the potential for future lineage diversification. While adult movements may contribute to range shifts, because of the strong natal philopatry exhibited by these species, we suggest that juvenile dispersal may better explain their global distribution and ocean basin colonization events.

Genetic analyses to date have identified well‐resolved relationships among the seven extant sea turtle species: a Carettini group including loggerheads (*Caretta caretta*), hawksbills (*Eretmochelys imbricata)*, and the ridleys (*Lepidochelys olivacea* and *L. kempii*); a Chelonini group including green turtles (*Chelonia mydas*) and flatbacks (*Natator depressus*); and a separate Dermochelyidae lineage of leatherbacks (*Dermochelys coriacea*) (Bowen & Karl, 2007; Duchene et al., 2012; Naro‐Maciel et al., 2008). These deeply diverged lineages diversified across ocean basins, with most species broadly distributed while others (*L. kempii* and *N. depressus*) are limited to one basin (Bowen & Karl, 1996). Previous studies examined relationships among sea turtle species and ocean basins through analyses of mtDNA and nuclear markers (Baltazar‐Soares et al., 2020; Bowen & Karl, 2007; Naro‐Maciel et al., 2008), while more recent whole mitogenome analyses increase molecular resolution, but are limited by small sample sizes (Cho et al., 2018; Duchene et al., 2012; Otálora & Hernández‐Fernández, 2018; Vilaça et al., 2021). In each case, genetic analyses focus almost exclusively on rookery sites and data, while the mechanisms driving diversification patterns may actually be due to misdirected philopatry among post‐dispersal individuals. Therefore, juvenile sea turtle dispersal in the context of the global gene tree may be key to understanding how and when populations established in each ocean basin, with ‘errors’ in natal philopatry post‐dispersal facilitating invasion into new basins and subsequent diversification (Bowen & Karl, 2007). However, in situ data on juvenile dispersal is lacking, mainly due to the difficulty of observing and sampling the early life stage, which for most species occurs far from shore over many years in an environment that is in constant motion. Further, published observations and samples of dispersal‐stage juveniles to date are mostly in the Atlantic basin (Bolten et al., 1998; Putman & Mansfield, 2015; Shamblin, Witherington, et al., 2018; Witherington, 2002; Witherington et al., 2012).

Within the Atlantic, there is high potential for multiple species and stocks to mix in the Gulf of Mexico, as ocean currents pass in close proximity to major rookeries throughout the basin and oceanic habitats within the Gulf occur relatively close to shore. These conditions present a unique opportunity to sample turtles in this elusive life stage (Putman & Mansfield, 2015; Shamblin, Witherington, et al., 2018; Witherington et al., 2012). Five of the seven sea turtle species are commonly found in the Gulf of Mexico at various life stages, including the Atlantic‐only Kemp's ridley (Valverde & Holzwart, 2017). Dispersal‐stage juveniles in the Gulf are likely a mix from source rookeries in the Gulf of Mexico, Caribbean, and Atlantic, and these juveniles may then continue dispersing via the Gulf Stream to the North Atlantic or the Mediterranean. Samples from this region can thus shed light on both past and future patterns of diversification within and among species.

To investigate juvenile sea turtle dispersal as a mechanism of connectivity, the goals of our study were to (1) identify the lineages represented in dispersal‐stage juvenile sea turtles in the Gulf of Mexico; (2) estimate the green turtle source rookeries contributing to the region, and (3) update the global gene tree of marine turtle mtDNA to refine our understanding of within‐species relationships and identify gaps in sampling across ocean basins and life stages. We present new haplotype data from dispersing sea turtles sampled in the Gulf of Mexico in a mixed stock analysis to estimate potential rookery contributions. We also present comprehensive curated long‐fragment haplotype data from the literature along with associated life‐stage and location metadata to reconstruct a mitochondrial haplotype tree representing global lineages from the seven extant species of sea turtles, a resource we hope other researchers will build upon in future analyses.

## 2 MATERIALS AND METHODS

### 2.1 Field sampling

**FIGURE 1:**
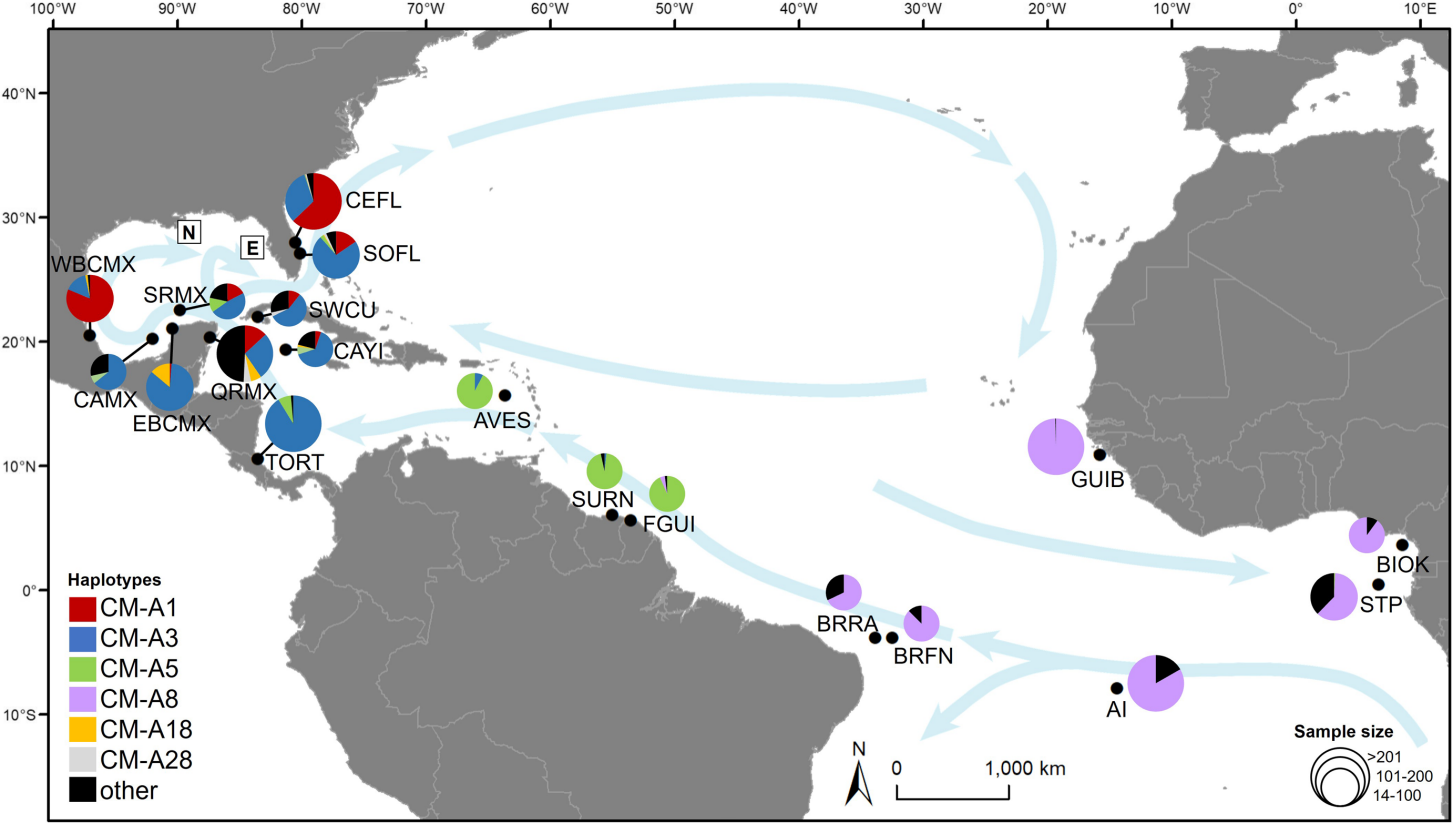
Dispersal‐stage juvenile green turtles were sampled from two areas, one in the Northern Gulf of Mexico (N; *n* = 20) and one in the Eastern Gulf of Mexico (E; *n* = 10). The locations of green turtle rookeries included in the mixed stock analysis are shown (black dots) along with their haplotype frequencies as reported in the literature (Barbanti et al., 2019; Bjorndal et al., 2005, 2006; Encalada et al., 1996; Formia et al., 2006, 2007; Hancock et al., 2019; Jordão et al., 2015; Millán‐Aguilar, 2009; Patrício et al., 2017; Pérez‐Ríos, 2008; Ruiz‐Urquiola et al., 2010; Shamblin et al., 2015, 2017; Shamblin, Bjorndal, et al., 2012; Shamblin, Witherington, et al., 2018; see Table S1). All hawksbills (*n* = 3) and loggerheads (*n* = 2) were encountered in the Northern Gulf of Mexico sampling area. The major currents are represented by blue arrows.

We sampled three species of dispersal‐stage juvenile sea turtles offshore in the northern and eastern Gulf of Mexico in 2013–2017. We launched 1 to 3‐day sampling trips annually from Venice, Louisiana, USA, with additional trips out of Cortez, Florida, USA, in 2016 (Figure 1). Samples from the Venice launch site are hereafter referred to as the Northern Gulf of Mexico, and samples from the Cortez launch site as the Eastern Gulf of Mexico (Figure 1). Each sampling trip occurred 25–120 km from shore in oceanic habitats. To locate these oceanic juvenile turtles, we first searched for floating lines of *Sargassum* seaweed and then navigated along the habitat in search of turtles on and around the *Sargassum* (Putman & Mansfield, 2015). Once a turtle was spotted, the vessel approached the turtles which we captured with a modified long‐handled dip net. In addition to recording standard morphometrics (e.g., carapace measurements, weight, head width), we sampled blood and/or skin from each turtle, after which we released them in *Sargassum* near the point of capture. We spun the blood samples to separate the plasma and used the red blood cells for subsequent genetic analyses. We placed skin samples in ethanol until analysis. All animal handling followed our Institutional Animal Care and Use Committee guidelines and was conducted under National Marine Fisheries Service permits 19508, 16733, and 1551.

### 2.2 DNA amplification and sequencing

We extracted DNA from 35 blood or skin samples using Qiagen DNeasy Blood & Tissue Kit standard protocols. From each DNA extraction, we amplified an ~800‐base pair fragment of the mitochondrial control region using the primer pair LCM15382 and H950 (Abreu‐Grobois et al., 2006) for the three hawksbills and two loggerheads. We amplified a longer ~950‐bp control region fragment in the 30 green turtle samples using the primer pair LCM15382 and CM16437 (Shamblin, Bjorndal, et al., 2012). These primers add 150 bp to the fragment obtained using LCM15382 & H950, which could increase the discrimination between haplotypes (Shamblin, Bjorndal, et al., 2012). Each 20 μl reaction contained 1 μl of DNA extract, 1 μl of each 10 μM primer, 2 μl 10× PCR buffer solution, 0.5 μl 2.5 mM dNTPs, 1.2 μl 25 mM MgCl_2_, 0.2 μl Taq DNA polymerase, and 13.1 μl water. The final concentrations were: 0.5 μM per primer, 10 mM Tris HCl pH 9.0, 50 mM KCl, 0.25 mM of each dNTP, 1.5 mM MgCl_2_, and 1 unit of Taq. For the primer pair LCM15382‐H950, the PCR cycling parameters we used were: 95°C for 3 min; 35 cycles of 95°C for 30 s, 55°C for 60 s, 72°C for 30 s; and then 72°C for 10 min. The PCR protocol for the primer pair LCM15382‐CM16437 was nearly identical to Shamblin, Bjorndal, et al. (2012) but with a slightly higher annealing temperature: 95°C for 5 min; 40 cycles of 95°C for 30 s, 57°C for 30 s, 72°C for 80 s; and then 72°C for 10 min. We purified each PCR product with ExoSAP‐IT™ following the manufacturer's protocol and sent them to Eurofins Genomics LLC for Sanger sequencing. For green turtles with the haplotype CM‐A1.1, we sequenced an additional ~300‐bp fragment from the ND5 region of the mtDNA with primers CM12751F and CM13064R (Shamblin et al., 2017) and the first PCR protocol listed above. This fragment was identified to contain a diagnostic SNP in a previous mitogenome study (Shamblin et al., 2017). We aligned, edited, and compared sequences to known Atlantic haplotypes in Geneious R9 software (Kearse et al., 2012).

### 2.3 Mixed stock analysis

Due to low sample sizes in two of the species, we focused on green turtles for a Bayesian mixed stock analysis (MSA) of individuals sampled in 2016–2017 using the *mixstock* package in R version 4.0.2 (Bolker et al., 2003, 2007; R Core Team, 2016) to estimate probabilities of source rookery contributions. We used the holistic “many‐to‐many” approach, which estimates contributions from potential source rookeries to multiple mixed destinations (Bolker et al., 2007), as opposed to the “many‐to‐one” model that estimates contributions to a single mixed site at time (Bolker et al., 2003; Pella & Masuda, 2001; Pella & Milner, 1987; Smouse et al., 1990). In addition to more closely reflecting sea turtle population connectivity, the “many‐to‐many” approach produces tighter confidence intervals than the “many‐to‐one” analysis (Bolker et al., 2007; Jensen et al., 2020). The *mixstock* package also computes an “unknown” mixed stock, without assuming rookeries contribute only to sampled sites (Bolker et al., 2007). We limited our mixed stock analyses to the dispersal stage for which we defined two potential mixed stocks: one in the Northern Gulf of Mexico and one in the Eastern Gulf (Figure 1). For the MSA, we truncated our sequences to shorter fragments (~500‐bp) to match the majority of rookery haplotype frequencies reported in the literature based on the shorter fragment.

The green turtle rookeries included in the analysis (Figure 1) were located along the coasts of Bijagós Archipelago, Guinea‐Bissau (GUIB); Bioko Island, Equatorial Guinea (BIOK); São Tomé and Príncipe (STP); Ascension Island (AI); Rocas Atoll, Brazil (BRRA); Fernando de Noronha, Brazil (BRFN); Awala‐Yalimapo and Cayenne, French Guiana (FGUI); Matapica and Galibi, Suriname (SURN); Aves Island, Venezuela (AVES); Tortuguero, Costa Rica (TORT); Grand Cayman, Cayman Islands (CAYI); Guanahacabibes Peninsula and San Felipe, Cuba (SWCU); Quintana Roo, Mexico (QRMX); Cayo Arcas, Mexico (CAMX); Scorpion Reef, Mexico (SRMX); Campeche and Yucatán, Mexico (EBCMX); Tamaulipas and Veracruz, Mexico (WBCMX); Jupiter Island, Tequesta, Singer Island, Boca Raton, Broward, Key West NWR, and Dry Tortugas, USA (SOFL); and Cape Canaveral, Melbourne Beach, and Hutchinson Island, USA (CEFL). We ran several mixed stock models that incorporated (1) rookery size, measured as the number of nests per year, and (2) probability of transport to the area by ocean currents (Bolker et al., 2007; Okuyama & Bolker, 2005; Putman & Mansfield, 2015).

Model 1 estimated rookery contributions by incorporating the haplotype frequencies from each potential source and both offshore sites along with the size of each rookery (Tables S1 and S2). We sourced rookery sizes from the literature (Bellini et al., 2013; Blumenthal et al., 2021; Broderick et al., 2006; Girard et al., 2016; Millán‐Aguilar, 2009; Rodríguez‐Martínez et al., 2021; Seminoff et al., 2015; Shamblin et al., 2015; Shamblin, Witherington, et al., 2018; van der Zee et al., 2019; Vera & Buitrago, 2012) to represent nest counts as close to the sampling period as possible given recent increases in green turtle rookery sizes at many sites (Seminoff et al., 2015). Model 2 also included particle back‐tracking probabilities from rookeries to the sampled area as calculated by Putman et al. (2015). Models 3 and 4 were similar to Models 1 and 2 but with the addition of haplotypes from dispersal‐stage green turtles sampled at similar sites to our Northern Gulf mixed stock samples as part of a separate study in 2009–2015 (Shamblin, Witherington, et al., 2018; Table S3). The sizes of the dispersing green turtles at our sites (Putman & Mansfield, 2015; current study) indicate that the turtles we encountered were likely 1–3 years of age (Reich et al., 2007; Witham & Futch, 1977). Therefore, Models 2 and 4 utilized particle back‐tracking probabilities within 2 years of drift to the sampled area (Putman et al., 2015) to scale rookery inputs to include transport probabilities (Okuyama & Bolker, 2005; Table S4). Models 2 and 4 did not include South Florida or Central Florida as potential source rookeries because the estimated probability of transport via ocean currents to the sample sites within 3 years is zero (Putman et al., 2015). Each model run consisted of 100,000 iterations with a burn‐in of 50,000. We ran the Gelman and Rubin shrink factor diagnostic to test for convergence (<1.2) (Pella & Masuda, 2001).

### 2.4 Global haplotype curation

To place the offshore juveniles sampled in the Gulf of Mexico in a broader phylogenetic context, we curated the named long‐fragment control region haplotypes for each of the seven sea turtle species found globally through a literature search and sequence similarity search on GenBank (Clark et al., 2016). For the literature search, we used Google Scholar to find studies that used the long‐fragment primers LCM15382/H950, LTEi9/H950 (Abreu‐Grobois et al., 2006), or the green turtle‐specific pair LCM15382/CM16437 (Shamblin, Bjorndal, et al., 2012), and downloaded sequences as provided by the authors or from GenBank accession IDs. For the GenBank search, we used BLAST (Clark et al., 2016) to find highly similar sequences to known haplotypes. In the case of Atlantic *C. caretta* and Atlantic *C. mydas* haplotypes, we additionally drew from the curated haplotype database on the Archie Carr Center for Sea Turtle Research website (https://accstr.ufl.edu/resources/mtdna‐sequences). We used the guidelines set forth by Arantes et al. (2020) to resolve redundancies in hawksbill haplotype naming. In the event that two or more haplotypes had different names but identical sequences, we collected the duplicate sequence names and retained the haplotype designation that was most consistent with others for the species. When two unique haplotype sequences were named identically, we appended the last name of the author who published the sequence in the literature or on GenBank. For each haplotype, we noted the life stage(s) and ocean basin(s) represented in the literature. We binned the life stages into five categories based on the size and location of encountered turtles: dispersal‐stage juveniles; post‐dispersal juveniles; mixed post‐dispersal juveniles/in‐water adults; in‐water adults; and rookery (from nesting female, egg, and/or hatchling samples). Some studies did not explicitly state which haplotypes belonged to which individuals sampled at mixed juvenile/adult foraging sites, which necessitated the mixed stage category. The in‐water adult observations consist of samples taken at foraging sites, and stranding data were assumed to occur near foraging sites.

### 2.5 Gene tree analysis

We imported sequences to Geneious and removed any duplicates. We used sequences from the alligator snapping turtle *Macroclemys temminckii* (EF071948.1) and the common snapping turtle *Chelydra serpentina* (EF122793.1) as outgroups. We aligned the sequences using the Clustal Omega algorithm with default parameters (Sievers et al., 2011) on the EBI server (Madeira et al., 2019) and manually adjusted the alignment in Geneious. To find the best model of sequence evolution, we used PartitionFinder v. 2.1.1 (Lanfear et al., 2017) and the Akaike Information Criterion for small sample sizes to select models of evolution to run in MrBayes. We ran PartitionFinder with both linked and unlinked branch lengths and used a greedy search algorithm. The greedy search algorithm uses a heuristic approach to search for a good partitioning scheme, as opposed to one that searches all possible partition schemes. The best model was the general time reversible model with invariant sites and gamma distribution of rates across sites (GTR + I + G). We reconstructed a Bayesian gene tree in MrBayes v. 3.2.7a on the CIPRES Science Gateway server (Miller et al., 2010) with two independent runs for 3.0 × 10^7^ generations and four chains each, sampling every 500th generation with the first 100,000 generations discarded as burn‐in. We confirmed Markov chain Monte Carlo convergence and adequate sampling of the posterior distribution (parameter ESS > 200) in Tracer v. 1.7 (Rambaut et al., 2018). We also reconstructed a maximum likelihood gene tree with the software IQ‐TREE on the IQ‐TREE web server (Trifinopoulos et al., 2016) to compare topologies. We visualized the Bayesian gene tree using the R package *ggtree* v3.3.0.900 in RStudio using R v. 4.1.2 (R Core Team, 2016; Yu et al., 2017) and incorporated the associated ocean basin and life‐stage data obtained during haplotype curation.

We used BEAST2 v. 2.6.6 (Bouckaert et al., 2019) on the CIPRES server to estimate divergence times between the sea turtle species and major lineages within species. We used the program BEAUTi (Drummond et al., 2012) to prepare the input file specifying the following parameters: the alignment, site model, clock model, MCMC chain length and sampling scheme, priors for the tree, and the birthrate and fossil calibration times. We implemented a strict clock and a Hasegawa‐Kishino‐Yano site model (Hasegawa et al., 1985), rather than the more parameter‐rich GTR used in the MrBayes analysis, to obtain chain convergence. We set a Yule tree prior (Yule, 1925) and one fossil calibration point at the root based on a divergence estimate between Dermochelyidae and Cheloniidae at 48.4–149.5 mya (Joyce et al., 2013) with uniform distribution.

We chose not to include three other fossil calibration points often cited in the literature. Recent studies adjust the Dermochelyidae‐Cheloniidae estimate, from >100 mya (Weems, 1988; Zangerl, 1980) to approximately 60 mya (Joyce et al., 2013; Shaffer et al., 2017; Thomson et al., 2021), which conflicts with fossil calibrations for Chelonini‐Carrettini at 50–75 mya (Ernst & Barbour, 1989; Weems, 1988) and suggests reexamination may also be needed for *Caretta*‐*Lepidochelys* at 12–20 mya (Carr & Marchand, 1942; Zangerl, 1980). The calibration point cited for divergence between *L. olivacea* and *L. kempii* (4.5–5 mya) is based on a single *L. kempii* fossil, which was dated indirectly (Dodd & Morgan, 1992), the use of which may artificially constrain divergence estimates.

We ran the BEAST analysis on the CIPRES server with a chain length of 1 × 10^8^, sampling every 10,000 generations and discarding the first 10,000,000 as burn‐in. We confirmed posterior distribution sampling in Tracer v. 1.7 as described for the previous analysis and calculated the final gene tree with divergence estimates and 95% highest posterior densities (HPD) in TreeAnnotator v. 1.2.59. We created a visualization of the resulting gene tree and divergence time estimates with the *ggtree* package in R (Yu et al., 2017).

## 3 RESULTS

### 3.1 Field sampling

We sampled and sequenced 35 dispersal‐stage turtles from three species in the northeastern Gulf of Mexico from 2013–2017: green turtles (*Chelonia mydas*, *n* = 30); hawksbills (*Eretmochelys imbricata*, *n* = 3); and loggerheads (*Caretta caretta*, *n* = 2). The sequenced turtle straight carapace lengths (SCL) ranged from 14.7–24.5 cm, with a mean size of 19.1 cm (SD 2.2 cm) for green turtles, 17.0 cm (SD 3.3 cm) for loggerheads, and 16.9 cm (SD 1.4 cm) for hawksbills. While sampled green turtles were larger on average, they were also the only species encountered during a late‐summer Eastern Gulf sampling trip in September 2016. Hawksbill and loggerhead encounters were limited to May–July in the Northern Gulf. We found four green turtle haplotypes: Cm‐A1.1 (*n* = 20); Cm‐A3.1 (*n* = 7); Cm‐A18.1 (*n* = 2); Cm‐A28.1 (*n* = 1). Of the individuals identified as Cm‐A1.1, we analyzed 19 for the diagnostic mitochondrial SNP, and all but one identified as Cm‐A1.1.1 (*n* = 18), with one Cm‐A1.1.2 (Shamblin et al., 2017). All three hawksbills sampled were the haplotype Ei‐A23. The two loggerheads sampled were Cc‐A1.1 and Cc‐A4.1 (Table S5).

### 3.2 Mixed stock analysis

**FIGURE 2:**
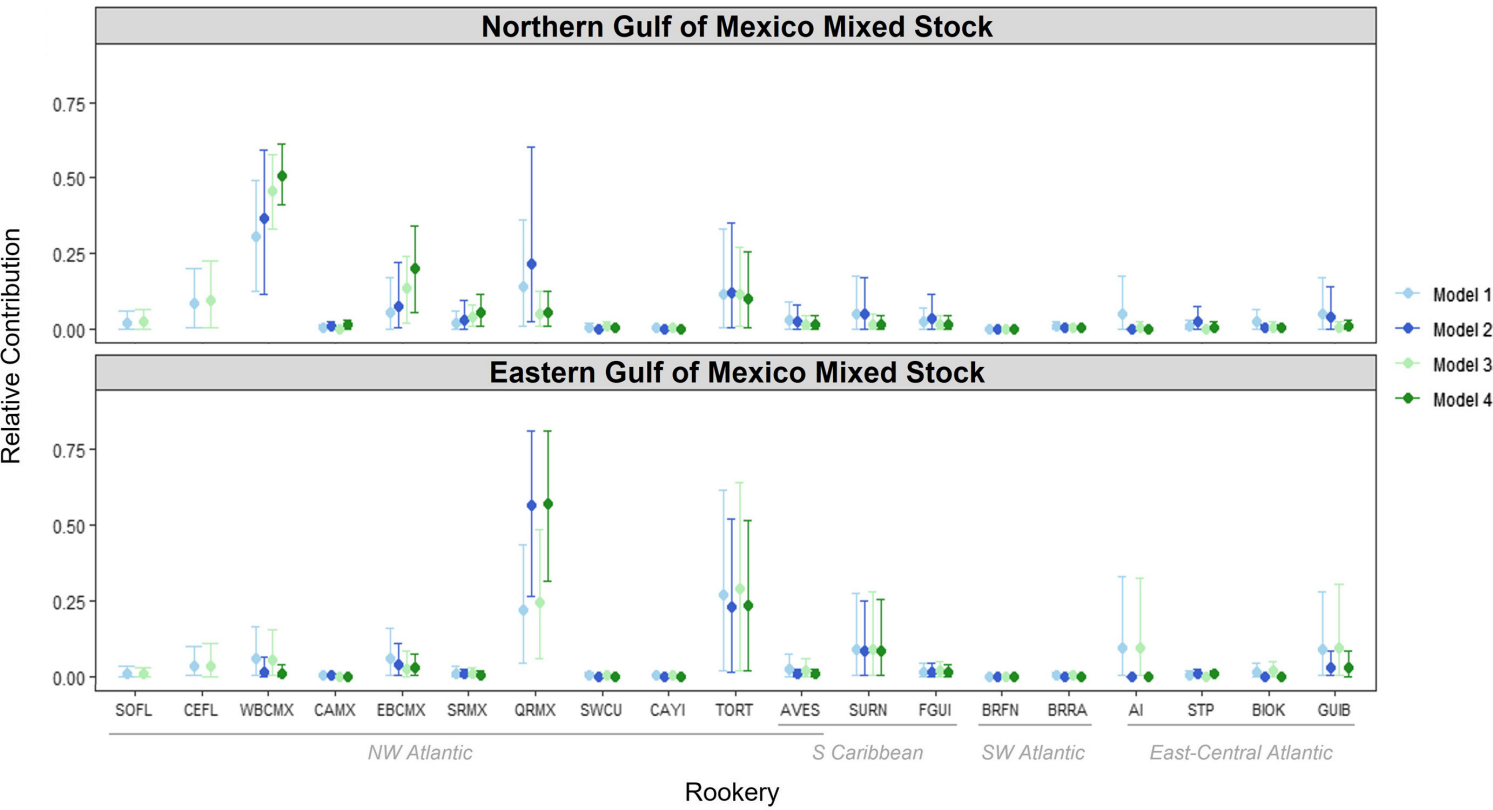
Mixed stock analyses for offshore juvenile green turtles sampled in two regions in the Gulf of Mexico. Models 1 and 2 include turtles sampled for the current study (Northern Gulf *n* = 20; Eastern Gulf *n* = 10), while Models 3 and 4 in green also include results from 121 samples reported by Shamblin, Witherington, et al. (2018) in the northern gulf. Points are mean estimates and whiskers indicate 95% credibility intervals. Rookeries along the *x*‐axis are grouped by regional management units (Wallace et al., 2010). In the most comprehensive Model 4, the highest estimated contributions to the Northern Gulf of Mexico were from rookeries along the Western Bay of Campeche (WBCMX: 0.51 [0.41–0.61]), Eastern Bay of Campeche (EBCMX: 0.20 [0.06–0.34]), and from Tortuguero, Costa Rica (TORT: 0.10 [0.00–0.26]), while the highest estimated contributions to the Eastern Gulf of Mexico originated from Quintana Roo, Mexico (QRMX: 0.57 [0.31–0.81]), Tortuguero (TORT: 0.23 [0.02–0.52]), and rookeries in Suriname (SURN: 0.09 [0.00–0.25]).

The MSA estimates from the four models indicated contributions from rookeries throughout the northwest Atlantic, Caribbean, and South Atlantic (Figure 2). The contributions to each offshore sampling area differed, with the Northern Gulf site receiving a higher proportion of juveniles from rookeries along the Western Bay of Campeche (Tamaulipas and Veracruz, Mexico) while there is a higher probability of rookeries along the eastern coast of the Yucatan (Quintana Roo, Mexico) contributing to the Eastern Gulf site. The credibility intervals around the estimates were reduced for the Northern Gulf in Models 3 and 4, while credibility intervals were broad for all models in Eastern Gulf, likely due to low sample sizes. General trends were similar across all four models (Table S6), and we will focus on the estimates from Model 4 here, which included transport probabilities, as well as additional samples from Shamblin, Witherington, et al. (2018). For the Northern Gulf, Model 4 indicates high contribution probabilities from three rookeries in Mexico (West Bay of Campeche, East Bay of Campeche, Quintana Roo), as well as from Costa Rica and Suriname (Figure 2). In contrast, the Eastern Gulf estimates suggest the highest contribution from Quintana Roo, with lower contributions from Costa Rica and Suriname.

### 3.3 Gene tree analysis

**FIGURE 3:**
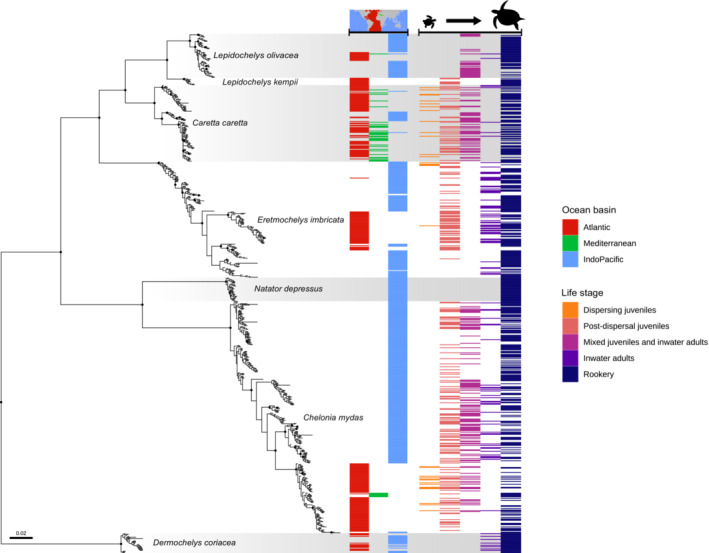
Bayesian gene tree of the seven extant sea turtle species based on long‐fragment mitochondrial DNA haplotypes. Reconstructed in the program MrBayes with ocean basins and sea turtle life stages in which each haplotype has been observed noted by bars to the right. Black dots on nodes indicate posterior probability support ≥0.99.

**FIGURE A1:**
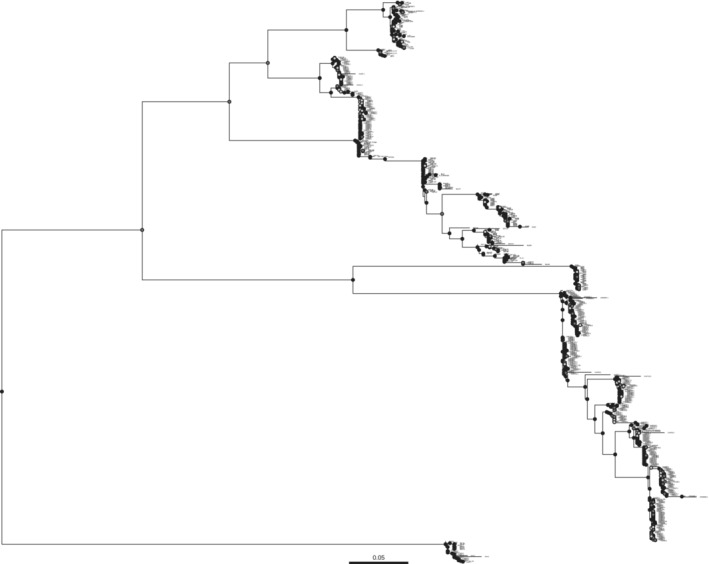
Maximum likelihood tree reconstructed in IQ‐TREE.

We assembled 709 unique long‐fragment mtDNA haplotype sequences across ocean basins and life stages (Tables S7–S14). The Bayesian and maximum likelihood gene trees produced similar topologies (Figure 3, Figure A1). Relationships among species were consistent with previous work (Bowen & Karl, 2007; Cho et al., 2018; Duchene et al., 2012; Evers & Benson, 2019; Naro‐Maciel et al., 2008; Otálora & Hernández‐Fernández, 2018). However, some within‐species relationships differed.

**FIGURE A2:**
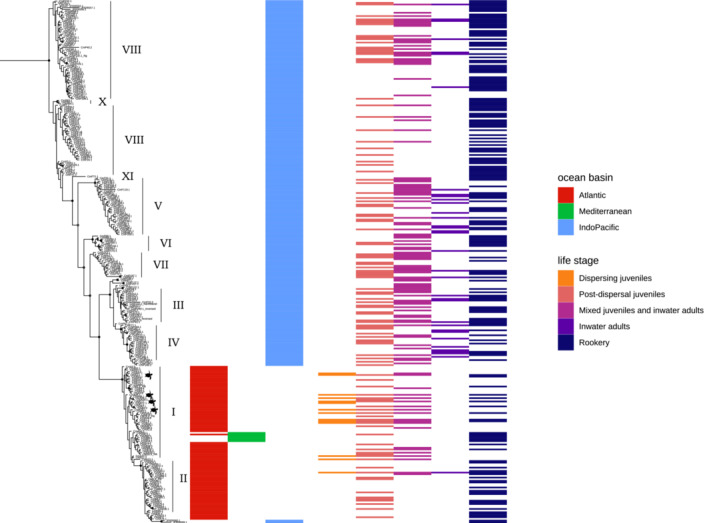
Green turtle clades from the Bayesian gene tree analysis. Major clades labeled as defined by Jensen et al. (2019). The haplotypes from dispersal‐stage juveniles we found in the Gulf of Mexico (*n* = 30) all fall within Clade I: Cm‐A1.1, Cm‐A3.1, Cm‐ A18.1, and Cm‐A28.1, denoted by the turtle icons.

The Atlantic/Mediterranean‐associated green turtle clades I and II were most closely related to Pacific clades III and IV as has been previously described (Jensen et al., 2019); however, the Atlantic clades were nested within other Pacific clades (Figure 3, Figure A2) in contrast with other studies, which found that the Atlantic clades split from Pacific clades closer to the root of the green turtle tree (Boissin et al., 2019; Duchene et al., 2012; Jensen et al., 2019). Unlike Jensen et al. (2019), the earliest green turtle split we identified was clade VIII from the rest of the clades with high confidence (posterior probability = 1). Two green turtle haplotypes (JF926559.1, JF926560.1) from the Indo‐Pacific rookery on Vamizi Island, Mozambique (Anastácio et al., 2014), fall within the Atlantic clade II with haplotypes from Brazil and Guinea‐Bissau (Patrício et al., 2017; Shamblin et al., 2015). Mediterranean green turtle haplotypes cluster with haplotypes in clade I found in the USA, specifically rookeries in the US Virgin Islands and Florida (Shamblin et al., 2015, 2017; Shamblin, Bjorndal, et al., 2012) and juveniles in Florida and Puerto Rico (Chabot et al., 2021; Gorham et al., 2016; Naro‐Maciel et al., 2017; Patrício et al., 2017). We identified just one green turtle haplotype found in both Atlantic and Mediterranean rookeries: CmA‐13.1 (Bradshaw et al., 2018; Garofalo et al., 2013; Gorham et al., 2016; Shamblin et al., 2015, 2017).

**FIGURE A3:**
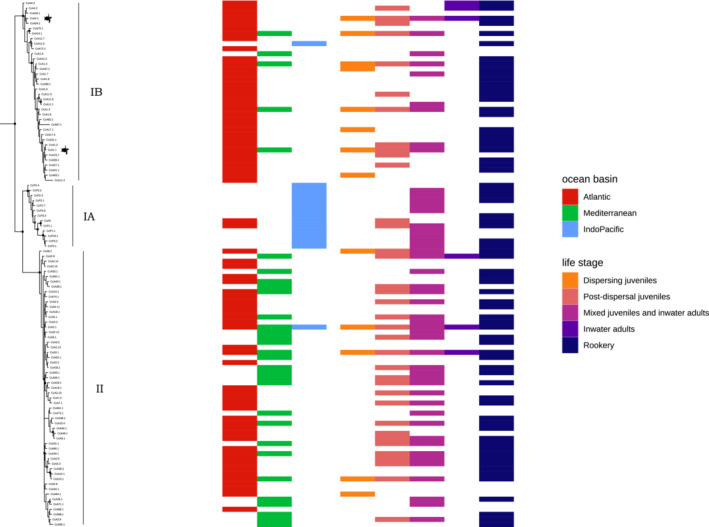
Loggerhead clades from the Bayesian gene tree analysis. Major clades labeled as defined by Shamblin et al. (2014). The haplotypes from dispersal‐stage juveniles we found in the Gulf of Mexico (*n* = 2) fall within Clade IB: CcA1.1 and CcA4.1, denoted by the turtle icons.

In loggerheads, Atlantic haplogroup II and Pacific haplogroup IA are more closely related to one another than either are to Atlantic haplogroup IB (Figure A3), unlike previous studies pairing IA and IB (Shamblin et al., 2014).

**FIGURE A4:**
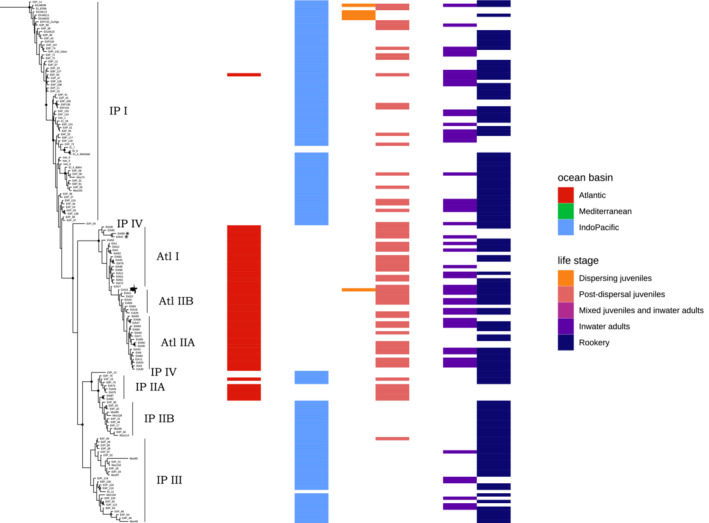
Hawksbill clades from the global Bayesian gene tree analysis. Major clades labeled as defined by Arantes et al. (2020). Asterisks (*) denote haplotypes in a different clade in our analyses than previous studies (EiA89 previously in Clade Atlantic IIA, EiA42 previously in Clade Atlantic IIB). The haplotype from dispersal‐stage juveniles we found in the Gulf of Mexico (*n* = 3), EiA23, denoted by the turtle icon in Clade Atlantic IIB.

In hawksbills, the Atlantic clades I, IIA, and IIB appear nested within the Indo‐Pacific clades. The “EiA” haplotypes within Indo‐Pacific clade II (EiA49, 70, 75, 82, and 87) are orphan haplotypes found in juveniles in the south Atlantic, likely of Indo‐Pacific origin because of close relationships with sequences from rookeries in Seychelles, Mozambique, and Chagos Archipelago (Figure A4).

**FIGURE A5:**
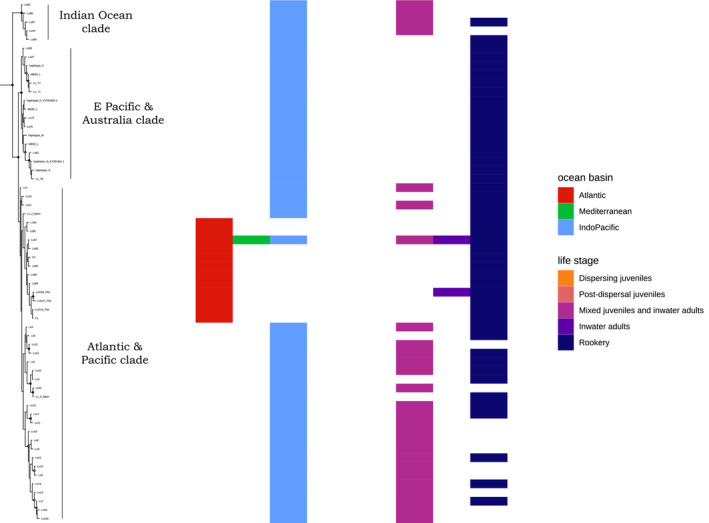
Olive ridley clades from the Bayesian gene tree.

**FIGURE A6:**
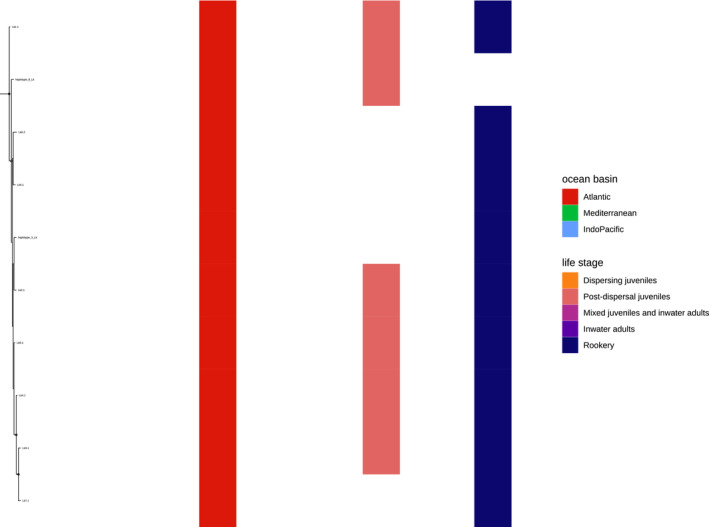
Kemp's ridley portion of the Bayesian gene tree.

**FIGURE A7:**
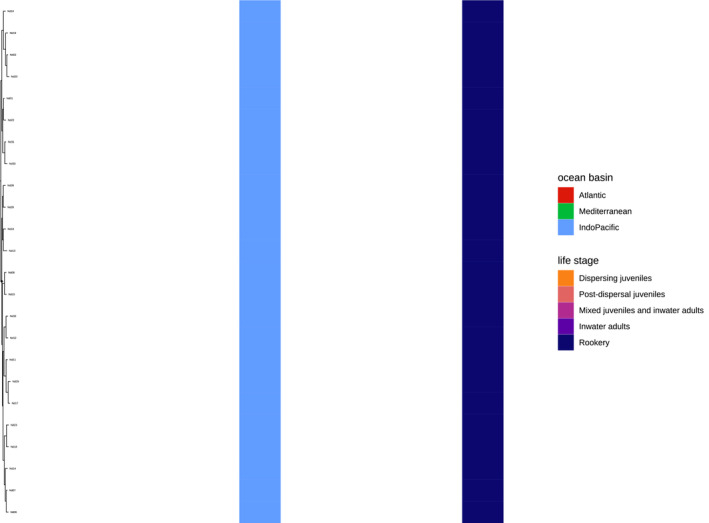
Flatback portion of the Bayesian gene tree.

**FIGURE A8:**
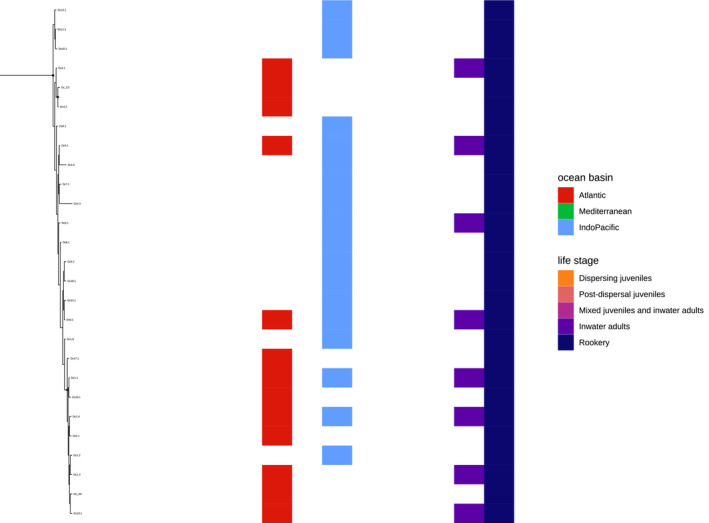
Leatherback portion of the Bayesian gene tree.

In terms of the life stages represented, we did not find long‐sequence mtDNA data for dispersal‐stage juvenile olive ridleys, Kemp's ridleys, flatbacks, or leatherbacks (Figure 3, Figures A5, A6, A7, A8).

### 3.4 Divergence estimates

**FIGURE 4:**
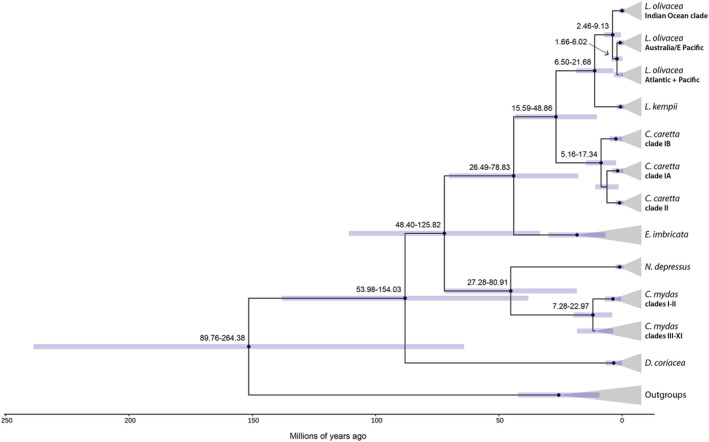
Dated Bayesian sea turtle mtDNA gene tree based on a Hasegawa‐Kishono‐Yano substitution model in BEAST. The bar at each node indicates the 95% highest posterior density interval. Black dots indicate nodes with posterior probability support ≥0.99. The pairing of *C. caretta* clades IA and II (posterior probability = 1) differs from Shamblin et al. (2014). This analysis also suggests an early division between *C. mydas* Atlantic clades I‐II and Pacific clades III‐XI (nomenclature from Jensen et al., 2019); however, this topology differs from the GTR gene tree in Figure 3.

**FIGURE A9:**
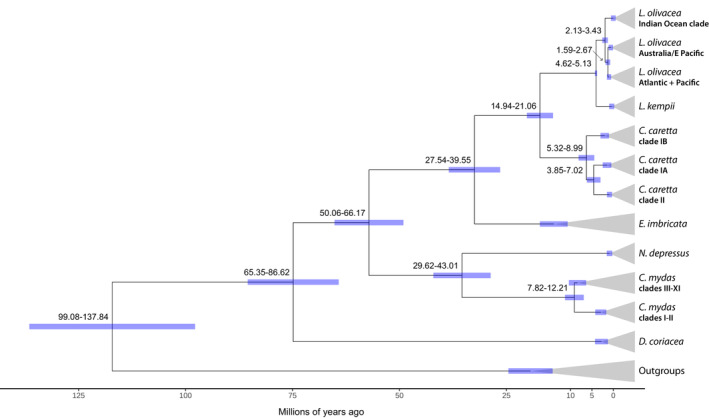
Results of the strict clock Bayesian model with four fossil calibration points commonly cited in the literature: Dermochelyidae–Cheloniidae 100–150 Ma (Weems, 1988; Zangerl, 1980), Chelonini‐Carrettini 50–75 Ma (Ernst & Barbour, 1989; Weems, 1988), *Caretta*–*Lepidochelys* 12–20 Ma (Carr & Marchand, 1942; Zangerl, 1980) and *L. olivacae*‐*L. kempii* 4.5–5 Ma (Dodd & Morgan, 1992; Hendrickson, 1980).

The following divergence time estimates are from the strict clock Bayesian model with a single calibration point, though we report estimates from a model with four common fossil calibrations in the Appendix (Figure A9) for comparison with other studies. Estimated divergence times at the species level were as follows: snapping turtle outgroup—marine turtles 152.73 mya; Dermochelyidae—Cheloniidae 89.34 mya; Carettini—Chelonini 73.40 mya; *Chelonia*—*Natator* 46.46 mya; *Eretmochelys*—*Caretta/Lepidochelys* 45.29 mya; *Caretta*—*Lepidochelys* 28.12 mya; and *L. olivacea*—*L. kempii* 5.14 mya (Figure 4).

Species divisions between ocean basins for hawksbills and leatherbacks were not well‐supported; therefore, divergence times are not presented below the species level for those species or the single‐basin Kemp's and flatbacks (Figure 4). Divergence estimates within the remaining species in Figure 4 reflect posterior probabilities of 99% or higher. The olive ridley Indian Ocean clade diverged at 5.14 mya (2.46–9.13); the remaining olive ridley clades split later at 3.37 mya (1.66–6.02).

The sorting of loggerhead clades IA and II as sister clades in the gene tree analysis was also well‐supported in the time tree. The divergence estimate between loggerhead clades II/IA from IB was 9.84 mya (5.16–17.34) followed by the split between clade II and Pacific IA at 7.44 mya (3.76–13.19). These divergence estimates among loggerhead clades are earlier than the divergence between the two *Lepidochelys* species estimated at 5.14 mya (6.50–21.68).

The chronogram suggests a split between Atlantic green turtle clades I and II from the Pacific clades at the root of the *C. mydas* clade with high support (posterior probability = 1). Interestingly, our gene tree nested Atlantic clades I and II within the Indo‐Pacific clades and paired with clades III and IV (Figure 3). With this in mind, our Atlantic‐Pacific lineage split should be interpreted with caution: our estimate of 13.14 mya (7.28–22.97) occurs much earlier than previous estimates of the split between clades I–II and clades III–IV at 2.34 mya (Jensen et al., 2019), 1.5–3 mya using RFLP mtDNA (Bowen et al., 1992), and 3.09 mya using whole mitogenomic sequences (Duchene et al., 2012), though closer to the 7.0 mya estimate that used a combination of nuclear and mtDNA sequences (Naro‐Maciel et al., 2008). Within Atlantic greens, the estimate for the split between clades I and II is 5.00 mya (2.41–9.00), again much earlier than Jensen et al. (2019) at 0.79 mya.

## 4 DISCUSSION

Our results fill in part of the sea turtle juvenile dispersal picture and illustrate the remaining data gaps. The comprehensive gene tree analysis of long‐fragment mtDNA shows considerable missing data for dispersal‐stage juveniles across basins, as well as post‐dispersal juveniles (Figure 3). These two life stages in particular need more sampling and monitoring; because of these species' long generation times, perturbations in the juvenile stages result in downstream population effects that may not be observable at rookeries for decades. Models of dispersal based on ocean currents alone can be used to build hypotheses for areas where juveniles will occur (Putman et al., 2015; Putman & Naro‐Maciel, 2013; Shamblin, Witherington, et al., 2018); however, the impact of turtle behavior on their ultimate paths (Putman & Mansfield, 2015) is still poorly understood and needs additional data from in situ sampling for ground‐truthing (Putman et al., 2016).

Our mixed stock analysis indicates that the majority of dispersal‐stage green turtles in the northeastern Gulf of Mexico originate in Mexico, though we should note that these analyses are based on available rookery data; estimates may change if more rookeries, higher sampling within each rookery, or longer DNA fragments are incorporated in the future. Putman et al. (2015) estimated that the oceanic juvenile green turtles impacted by the Deepwater Horizon oil spill in the northern Gulf of Mexico likely originated from Mexico, Costa Rica, Suriname, and Guinea‐Bissau based on ocean currents and rookery sizes. However, the combination of genetic evidence and current transport we present here suggests little if any contributions from Suriname and Guinea‐Bissau (Figure 3), likely due to limited overlap in haplotypes between our sites and these rookeries informing the MSA. Our data collected in 2016–2017 indicate that Quintana Roo, Mexico is the major contributor to the Eastern Gulf of Mexico sampling site, while a higher proportion of juveniles in the northern Gulf originate along the western Bay of Campeche. However, we note that the majority of the Eastern Gulf samples were from a later sampling trip in September 2016, and juvenile dispersal patterns may differ in early summer versus late summer due to shifts in the currents and hatching times. Turtles originating from Quintana Roo are likely to encounter the loop current, and based on its dynamics at the time of hatching, dispersing juveniles will either enter the Gulf of Mexico or bypass the Gulf and join the Gulf Stream at the southern tip of Florida and travel into the North Atlantic. The combination of our samples from the northern Gulf with the results of Shamblin, Witherington, et al. (2018) provides additional evidence that a majority of the dispersal‐stage green turtles in the northern Gulf of Mexico originate from the western Bay of Campeche, or that currents linking these two areas were stronger during the years sampled. A previous study of juvenile green turtle strandings along the coast of Texas, USA, also found rookeries along the western Gulf of Mexico as a likely source (Shamblin et al., 2017). Of the 19 Cm‐A1.1 green turtles that we analyzed for the additional diagnostic mitochondrial SNP, all but one matched the Cm‐A1.1.1 haplotype previously found to be fixed in samples analyzed from a western Bay of Campeche rookery (Shamblin et al., 2017). Together, these results indicate that the rookeries along the western Bay of Campeche are major contributors to the genetics of dispersal‐stage juvenile green turtles in the northern Gulf of Mexico.

Though a small green turtle rookery, juveniles from the Cayman Islands may complicate mixed stock estimates because of re‐introductions from outside rookeries (Costa Rica, Suriname, Guyana, Ascension Island) and in‐water sites (Costa Rica, Suriname, Guyana, Ascension Island, Mexico, and Nicaragua), which were collected to stock the Cayman Turtle Farm in the 1960s–70s (Barbanti et al., 2019). Subsequent releases of head‐started juveniles have been organized to replenish the natural population (Barbanti et al., 2022; Bell et al., 2005). Therefore, it is possible that haplotypes suggesting connectivity with Costa Rica and Suriname in our models are actually from the Cayman Islands. Additional sampling from this rookery will help clarify this issue. As conservation managers develop plans for future reintroduction initiatives, the tools are now available to better match the genetics of the recipient population to the source population.

We did not have sufficient sample sizes to perform mixed stock analyses for dispersal‐stage loggerheads and hawksbills in the Gulf of Mexico, and inferences about source populations are, therefore, limited. The EiA23 hawksbill haplotype has been considered by some exclusive to rookeries in Mexico (Labastida‐Estrada et al., 2019), and while it has also been found in rookeries in the Dominican Republic (Carreras et al., 2013) and US Virgin Islands (Leroux et al., 2012), its highest relative frequency occurs at Mexican rookeries along the Yucatan Peninsula (Labastida‐Estrada et al., 2019; Leroux et al., 2012). The connectivity between Mexican green turtle rookeries and dispersal‐stage juveniles in the Gulf of Mexico (Figure 2) may also occur in hawksbills, and is consistent with previous rookery estimates for coastal post‐dispersal juvenile hawksbills observed in the southeastern Gulf of Mexico (Gorham et al., 2014) and southeastern Florida (Wood et al., 2013); however, additional sampling of dispersal‐stage individuals in the Gulf is needed to confirm. If only using a short fragment, the long‐fragment haplotype EiA23 is indistinguishable from EiA24, EiA39, EiA41, EiA42, EiA43, and EiA83, found in Mexican rookeries but also in the Dominican Republic, Trinidad and Tobago, Antigua and Barbuda, Nicaragua, and Puerto Rico, USA (Carreras et al., 2013; Cazabon‐Mannette et al., 2016; Labastida‐Estrada et al., 2019; Leroux et al., 2012; Levasseur et al., 2019; Velez‐Zuazo et al., 2008), illustrating that the longer mtDNA fragment is key for higher genetic resolution among rookeries.

The two haplotypes found in the dispersal‐stage loggerheads in this study, Cc‐A4.1 and Cc‐A1.1, both fall within haplogroup IB (Figure A3). While Cc‐A1.1 is common at nearby rookeries along the southeastern US, Cc‐A4.1 has only been found in Brazilian rookeries to date (Shamblin et al., 2014). The long transport of this haplogroup is not an isolated event, as Cc‐A4 has been found in juveniles caught as bycatch in the North Atlantic “northeast distant” fisheries region (LaCasella et al., 2014; Stewart et al., 2019) and a North Carolina pound net fishery (Bass et al., 2004), as well as in a loggerhead‐green turtle hybrid encountered along the Florida coast (Shamblin, Mansfield, et al., 2018). Loggerhead juvenile dispersal to the North Atlantic from South Atlantic rookeries may be facilitated by seasonal shifts in the South Equatorial Current late in the Brazilian loggerhead hatching season, distributing hatchlings northward (Mansfield et al., 2017). This dispersal‐stage connectivity supports hypotheses that Cc‐A1.1 in the USA may stem from the Cc‐A4 lineage in Brazil (Baltazar‐Soares et al., 2020), as opposed to the Brazilian population established from the USA (Reis et al., 2010). Broad juvenile dispersal may be the key mechanism behind this lineage colonizing new regions and basins.

Assumptions about connectivity among populations and lineages are limited by the breadth and depth of sampling. For example, a study of stranded juvenile loggerheads along the coast of France concluded that turtles with the haplotype Cc‐A1.3 must have originated from Cape Verde, as it had only been observed in Cape Verdean rookeries at the time (Monzón‐Argüello et al., 2010, 2012). But that haplotype has since been sampled at rookeries in North America (Shamblin, Bolten, et al., 2012). One assumption of mixed stock models is that all source populations have been adequately sampled. Increasing sample sizes, sites, markers, and data sharing among studies will further improve future estimates.

Worldwide, the largest sampling gap across sea turtle species is the dispersal stage (Figure 3). Haplotypes for this life stage are so far only available from the Gulf of Mexico for green turtles (current study, Shamblin, Witherington, et al., 2018), the Gulf of Mexico (current study) and strandings in France (Monzón‐Argüello et al., 2012) for loggerheads, and the Gulf of Mexico (current study) and strandings in UAE (Natoli et al., 2017) for hawksbills. Connectivity among life stages is also difficult to characterize in a genetic framework because there are few nucleotide differences between mtDNA haplotypes, both for delineating within‐species lineages and among species for which close genetic relationships remain despite deep divergence.

**FIGURE A10:**
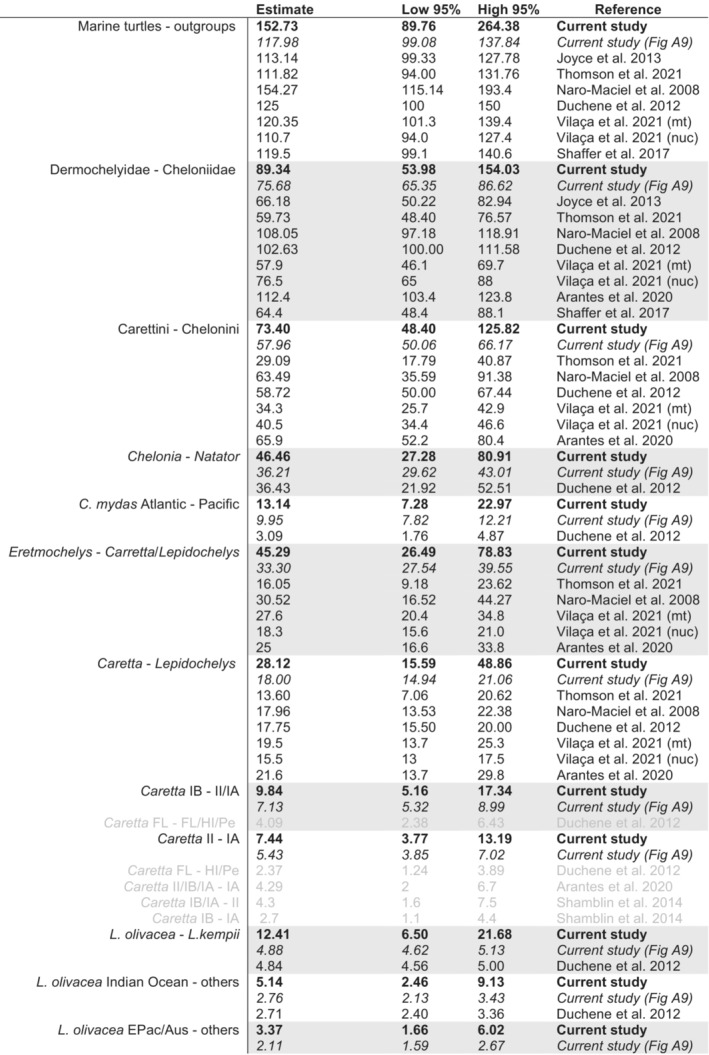
Divergence time estimates from the current study (bold and italics) and comparable estimates from the literature. Grey text indicates estimates for topologies that differed.

Previous estimates of species divergence times vary, generally 50–110 million years for the separation between leatherbacks and the hard‐shelled species, and 25–65 million years for dividing Carettini from Chelonini (Arantes et al., 2020; Duchene et al., 2012; Joyce et al., 2013; Naro‐Maciel et al., 2008; Thomson et al., 2021; Vilaça et al., 2021). Our marine turtle divergence estimate of 152.73 mya is similar to previous estimates for the split from the snapping turtle lineage (Figure A10). The emergence of Dermochelyidae at 89.34 mya is closest to a previous estimate based on nuclear DNA across the genome (Vilaça et al., 2021). At the shallower internal nodes, our estimates track closely with those based on an analysis of whole mitogenomes (Duchene et al., 2012). The consistency of our estimates with studies that include a range of nuclear and mitochondrial markers may be surprising given that we reconstructed a chronogram based on only a fragment of the mitochondrial genome, though it speaks to the utility of mtDNA fragments when used in large sample sizes.

Our gene tree analysis recovered the 11 green turtle clades previously described by Jensen et al. (2019), though with a longer mtDNA fragment and additional haplotypes our topology differs (Figure A2). The deep divergence of green turtle clade VIII suggests an Indo‐Pacific origin for the species, a hypothesis proposed for loggerheads, ridleys, and leatherbacks as well (Bolten et al., 1998; Dutton et al., 1999; Shamblin et al., 2014; Shanker et al., 2004). Like Jensen et al. (2019), our results from the gene tree analysis in MrBayes paired Atlantic clades I and II with Indo‐Pacific clades III and IV, while our dated tree results from BEAST split Atlantic clades I and II at the base of the green turtles. This difference may be because our dated tree is based on a HKY model while the MrBayes tree is based on a GTR model, and suggests that the more‐informative GTR‐modeled topology with the Atlantic clades nested within Indo‐Pacific clades may be more accurate. The Atlantic hawksbill clades are also nested within the Indo‐Pacific lineages in our gene tree analysis (Figure 3), suggesting a similar diversification pattern in both green turtles and hawksbills (Nishizawa et al., 2010, 2012; van der Zee et al., 2021). Within the mainly Indo‐Pacific hawksbill Clade IP‐I, the haplotypes EiIP‐27, EiIP‐33, and EiIP‐36 span opposite sides of the Indo‐Pacific from Iran, UAE, and Seychelles to the Pacific coast of central America (Gaos et al., 2016, 2018, 2020; LaCasella et al., 2014; Natoli et al., 2017; Tabib et al., 2014; Vargas et al., 2016; Zuñiga‐Marroquin & De Los Monteros, 2017; Table S10). Additionally, a juvenile hawksbill with the haplotype EiP‐33 observed off the coast of Brazil (Vilaça et al., 2013) is so far the only observation from Clade IP‐I in the Atlantic but demonstrates that connectivity through juvenile dispersal may have facilitated the establishment of the Atlantic clades from the Indo‐Pacific. On the other hand, evidence of connectivity between haplotypes from green turtle rookeries in Mozambique (Anastácio et al., 2014), which fall within Atlantic clade II (Figure 3, Figure A2), previously seen with short fragments (Bourjea et al., 2007), provides evidence of Atlantic to Indo‐Pacific movement more recently. Additional trans‐basin juvenile dispersal is evident in loggerheads, with Atlantic haplotypes CcA‐1.1, CcA‐1.3, CcA‐1.4 recovered from juveniles in the Mediterranean (Clusa et al., 2014; Garofalo et al., 2013; Tolve et al., 2018) and hawksbills, with Atlantic orphan haplotypes EiA‐49, EiA‐70, EiA‐75, EiA‐82, and EiA‐87 closely related to sequences from Indo‐Pacific rookeries in Seychelles, Mozambique, and Chagos Archipelago (Anastácio & Pereira, 2017; Monzón‐Argüello et al., 2011, 2010; Putman et al., 2014; Vargas et al., 2016; Vilaça et al., 2013; Figure A4).

The pairing we found of loggerhead clades IA and II differs from other recent analyses that paired Atlantic/Mediterranean clade IB with Pacific IA (Bowen, 2003; Shamblin et al., 2014) though is similar to an earlier study using short mtDNA fragments (Bowen et al., 1994). Based on a previous haplotype network analysis, the haplotypes in Pacific clade IA appear to cluster in an intermediate position with mutational steps in either direction to the two Atlantic/Mediterranean clades IB and II (Arantes et al., 2020). Our gene tree analyses contain more haplotypes from the Pacific clade IA compared to previous studies, which may explain the shifted pairing of sister clades. This arrangement of the clades (Figure 4, Figure A3) supports the hypothesis of two dispersal events from the Indo‐Pacific establishing the Atlantic lineages (Baltazar‐Soares et al., 2020). We echo a call by Shamblin et al. (2014) for additional sampling and deeper genetic analysis from two large Indo‐Pacific rookeries with only one mtDNA haplotype identified at each to date: Masirah Island, Oman, and Tongaland, South Africa. The position of each of these haplotypes (Cc‐A11.6 and Cc‐A2.1, respectively) nested in separate Atlantic clades may indicate more recent dispersal to the Indian Ocean from Atlantic (Bowen et al., 1994). The haplotypes from Mediterranean loggerhead rookeries are exclusive to Clade II, though juveniles from Clade IB have been observed in the Mediterranean (Clusa et al., 2014; Garofalo et al., 2013; Tolve et al., 2018) and may provide insight into future diversification. The longer estimated duration loggerhead juvenile dispersal stage—based on their larger size at recruitment to post‐dispersal habitats of ~55 cm as opposed to ~25 cm in green turtles and Kemp's ridleys (Bolten, 2003)—likely helps explain these disparate colonization waves.

The Gulf of Mexico is an important habitat for adult foraging leatherbacks from nesting beaches in Costa Rica and Panama (Evans et al., 2021); however, data are scarce for juvenile leatherbacks in the Gulf of Mexico, or any ocean basin, because of their exclusively offshore life history (Bolten, 2003). With similar sampling gaps for olive ridleys and flatbacks (Figure 3), collaboration with commercial and traditional fisheries (LaCasella et al., 2014; Lopez‐Mendilaharsu et al., 2019; Ng et al., 2014; Parker et al., 2005, 2011; Stewart et al., 2019) and local non‐profit groups will facilitate sample collection.

Our results highlight the potential role of juvenile dispersal in introducing founder events and subsequent diversification, particularly for migratory species with natal philopatry like sea turtles. Continued in‐water and rookery research projects across species, along with updated mixed stock analyses such as the current study, will further improve estimates of connectivity within and among life stages and ocean basins. In addition, standardized curation and cooperative management of haplotypes and other genetic datasets along with associated metadata are sorely needed. We urge fellow researchers to report long mtDNA fragment sequences, even if trimmed for MSA or other analyses for publication. We now have fully annotated leatherback and green turtle genomes (Bentley et al., 2022) that can be used to develop genome‐wide genetic datasets for many individuals, which will facilitate much more robust analyses of evolutionary history and population structuring. In the meantime, mtDNA data provide valuable insight into connectivity and patterns of diversification across habitats and life stages.

## AUTHOR CONTRIBUTIONS

**Katrina F. Phillips:** Conceptualization (lead); data curation (equal); formal analysis (equal); investigation (lead); project administration (lead); supervision (supporting); visualization (equal); writing – original draft (lead); writing – review and editing (lead). **Katherine R. Martin:** Data curation (equal); formal analysis (equal); investigation (supporting); visualization (equal); writing – review and editing (supporting). **Gustavo D. Stahelin:** Data curation (supporting); formal analysis (supporting); investigation (supporting); visualization (supporting); writing – review and editing (supporting). **Anna E. Savage:** Conceptualization (supporting); formal analysis (supporting); funding acquisition (supporting); supervision (supporting); writing – review and editing (supporting). **Katherine L. Mansfield:** Conceptualization (supporting); funding acquisition (lead); investigation (supporting); project administration (supporting); supervision (lead); writing – review and editing (supporting).

## CONFLICT OF INTEREST

The authors declare that there is no conflict of interest.

## Supporting information


Tables S1–S6

Tables S7–S14

## Data Availability

The data that support the findings of this study are openly available on GitHub at https://github.com/katherinermartin/Phillips_et_al_mtDNA.
